# Prognostic Differences Between Early-Onset and Late-Onset Colorectal Cancer

**DOI:** 10.3390/medicina61030390

**Published:** 2025-02-24

**Authors:** Vlad Alexandru Ionescu, Gina Gheorghe, Ioana-Alexandra Baban, Alexandru Barbu, Teodor Florin Georgescu, Loredana-Crista Tiuca, Ninel Antonie Iacobus, Camelia Cristina Diaconu

**Affiliations:** 1 Faculty of Medicine, University of Medicine and Pharmacy Carol Davila Bucharest, 050474 Bucharest, Romania; gina.gheorghe@drd.umfcd.ro (G.G.); florin.georgescu1@yahoo.com (T.F.G.); loredana-crista.laslo@drd.umfcd.ro (L.-C.T.); nine-iacobus.antonie@drd.umfcd.ro (N.A.I.); camelia.diaconu@umfcd.ro (C.C.D.); 2 Internal Medicine Department, Clinical Emergency Hospital of Bucharest, 105402 Bucharest, Romania; 3 Gastroenterology Department, Central Military Emergency University Hospital, 010825 Bucharest, Romania; ioana.baban99@gmail.com (I.-A.B.); alexandru.barbu212@gmail.com (A.B.); 4 General Surgery Department, Clinical Emergency Hospital of Bucharest, 105402 Bucharest, Romania; 5 Academy of Romanian Scientists, 050085 Bucharest, Romania

**Keywords:** early-onset colorectal cancer, late-onset colorectal cancer, prognosis, endoscopic diagnosis, histopathological diagnosis, prognosis

## Abstract

*Background and Objectives*: Early-onset colorectal cancer (EO-CRC) has become a significant public health concern due to its alarming rise in incidence and the poor prognosis associated with this disease. The aim of our study was to identify epidemiological, clinical, and paraclinical characteristics that could explain the more aggressive evolution of EO-CRC compared to late-onset colorectal cancer (LO-CRC). *Materials and Methods*: We conducted a retrospective study over a two-year period, including 204 patients diagnosed with colorectal cancer (CRC). The patients were divided into two subgroups: those with EO-CRC and those with LO-CRC. Statistical analysis was performed using IBM SPSS Statistics, Version 29.0. *Results*: EO-CRC was identified in 11.3% of the patients included in the study. Compared to LO-CRC patients, EO-CRC patients exhibited a tendency for more distal tumor localization and a stenotic endoscopic appearance (43.5% vs. 29.3%). Regarding histopathological diagnosis, EO-CRC patients demonstrated a higher proportion of the mucinous histologic subtype (34.8% vs. 14.4%) and a significantly greater percentage of poorly differentiated tumors (39.1% vs. 14.5%; *p* = 0.010). Immunohistochemical results, available for a limited number of patients, revealed higher CDX2 positivity in LO-CRC patients (*p* = 0.012) and higher HER2 positivity in EO-CRC patients (*p* = 0.002). Smoking (*p* = 0.006) and hypertension (*p* = 0.002) were more prevalent in EO-CRC patients than in LO-CRC patients. *Conclusions*: Patients with EO-CRC exhibit distinct histopathological and molecular characteristics compared to those with LO-CRC, which may contribute to their poorer prognoses. The higher prevalence of the mucinous histological subtype, poor tumor differentiation, increased HER2 expression, and reduced CDX2 expression suggest potential molecular pathways driving the aggressive nature of EO-CRC. These findings highlight the need for tailored screening strategies and personalized therapeutic approaches in younger CRC patients. Future studies should further investigate the underlying mechanisms and potential biomarkers that could guide early diagnoses and targeted treatments.

## 1. Introduction

Colorectal cancer (CRC) represents a significant global public health concern, ranking as the third most common cancer and the second leading cause of cancer-related mortality [1]. In 2020, CRC accounted for 10% of all global cancer cases (1,931,590 cases) and 9.4% of cancer-related deaths (935,173 deaths) [1]. According to the latest studies from the International Agency for Research on Cancer (IARC), the annual number of new CRC cases is projected to increase by 63% by 2040, reaching 3.2 million, while CRC-related mortality is expected to rise by 73%, reaching 1.6 million deaths per year during the same period [2]. Moreover, this increase in incidence rates has been observed to be more pronounced among young adults and in countries undergoing economic transitions [2]. In Romania, CRC ranks as the second most common malignancy and the second leading cause of cancer-related deaths in both sexes [3].

CRC incidence rates exhibit geographic variability, with a direct correlation to the Human Development Index (HDI) [4]. Countries with a high HDI have been shown to have a CRC incidence rate four times higher compared to those with a low HDI [4]. However, recent epidemiological data indicate a stabilization or even a decline in CRC incidence in developed countries, while an increasing trend has been observed in developing countries [4,5]. This phenomenon may be explained by the adoption of a Western lifestyle and increased exposure to specific CRC risk factors [4,5].

The implementation of screening programs has improved the early detection of CRC in patients over 50 years of age, contributing to reduced morbidity and mortality rates associated with this condition [6,7]. However, the proportion of patients with early-onset colorectal cancer (EO-CRC), defined as CRC diagnosed under the age of 50 years, has steadily increased over recent decades [7,8,9,10,11,12]. For instance, Yeo et al. reported an annual increase in CRC incidence among adults under 50 years of approximately 1.4% per year, alongside a decline in incidence among adults over 50 years of about 3.1% per year [11]. Additional studies predict that, within the next decade, 25% of rectal cancers and 10–12% of CRC cases will be diagnosed in individuals under the age of 50 years [13]. These alarming epidemiological trends have led to updates in CRC screening guidelines in the United States, lowering the recommended starting age for screening to 45 years [14].

From a molecular perspective, CRC diagnosed before the age of 50 years differs significantly from CRC diagnosed after this age in terms of oncogenic mutation frequencies, a higher prevalence of mucinous (poorly differentiated) histology, a distinct DNA methylation profile, more distal localization, and poorer survival rates [15,16,17]. Cancers developing in the distal colon and rectum are associated with a high intake of red and processed meat, a low intake of fish and poultry, and increased alcohol consumption [18].

However, despite the concerning increase in EO-CRC prevalence, there remains a lack of comprehensive observational and experimental studies exploring the factors that drive its distinct clinical course and poorer prognosis [18]. While existing research has identified certain molecular and histopathological differences between EO-CRC and LO-CRC, the extent to which these characteristics influence disease progression, response to treatment, and long-term outcomes remains insufficiently understood. Specifically, there is a limited understanding of how factors such as risk factors, tumor differentiation, biomarker expression, and comorbidities contribute to the aggressive nature of EO-CRC. Building on these gaps, we conducted a study aimed at comparing a series of demographic, clinical, and paraclinical variables between patients with EO-CRC and those with LO-CRC. The primary objective of our study was to identify key prognostic factors that may explain the more aggressive behavior of EO-CRC and inform targeted screening and treatment strategies to improve patient outcomes.

## 2. Materials and Methods

We conducted a retrospective observational study over a 2-year period (January 2021 to January 2023), including 204 patients admitted to the Clinical Emergency Hospital of Bucharest, Romania, with a diagnosis of CRC. The patients were divided into two main groups based on the age at diagnosis:

- Group 1—patients with EO-CRC (diagnosed at or before the age of 50 years);
- Group 2—patients with LO-CRC (diagnosed after the age of 50 years).

The study received ethical approval from the Ethics Committee of the Clinical Emergency Hospital of Bucharest (approval number 1400/7 February 2023). Additionally, all patients included in the study provided written informed consent, agreeing to the use of their personal data for medical education purposes. The inclusion criterion was a confirmed diagnosis of CRC based on histopathological examination. The exclusion criteria included the absence of signed informed consent, missing data relevant to the study in the medical records, or a diagnosis of cancer at another site. None of the patients initially included in the study were excluded at a later stage. The database for the study was created by reviewing patient records, including observation sheets, imaging results, and laboratory investigations available in the archives of the Clinical Emergency Hospital of Bucharest, in accordance with the inclusion and exclusion criteria. We also analyzed the results of histopathological examinations (including histologic type and differentiation grade) and immunohistochemical analyses, assessing the expression of various markers such as CDX2 (caudal-type homeobox transcription factor 2), HER2 (human epidermal growth factor receptor 2), Ki67 (antigen Kiel 67), MLH1 (MutL homolog 1), MSH2 (MutS homolog 2), MSH6 (MutS homolog 6), PMS2 (post-meiotic segregation increased 2), AE1/3 (cytokeratin AE1/AE3), CA 19.9 (carbohydrate antigen 19.9), CK20 (cytokeratin 20), CK7 (cytokeratin 7), DPC (DNA–protein cross-links), and ER (estrogen receptor).

### 2.1. Statistical Analysis

For statistical analysis, we used IBM SPSS Statistics for Windows, Version 29.0 (30-day trial version) from IBM Corp., Armonk, NY, USA. Nominal data were presented as absolute frequencies and percentages, while continuous variables were expressed as mean ± standard deviation (SD). Associations between categorical variables were analyzed using cross-tabulation and the chi-square (χ^2^) test. When chi-square results were significantly affected, rendering them unreliable, Fisher’s exact test was applied.

To compare means for dichotomous variables, the independent samples *t*-test was used. For comparisons involving three or more group means with repeated measurements on the same participants, the ANOVA test was employed. A *p*-value < 0.05 was considered statistically significant.

### 2.2. Simple Linear Regression

Regression analysis, as an application of correlation, was utilized for predictive purposes. This approach aimed to determine the linear relationship between variables, described through a mathematical equation. The primary goal was to predict the values of one variable based on the values of the other, using the regression equation. The linear relationship between two variables was examined via linear regression, with one designated as the independent variable (predictor) and the other as the dependent variable (outcome). The linear correlation was expressed through a regression equation, which geometrically corresponds to the regression line.

### 2.3. Multiple Linear Regression

The relationship between the dependent variable and statistically significant independent variables, as identified through simple linear regression analysis, was subsequently included in a multiple linear regression model.

### 2.4. Binomial Logistic Regression

Binomial logistic regression was employed to assess correlations between two categories of variables. To determine whether the probability of an event was similar between two distinct patient groups, the estimated relative risk (odds ratio, OR) was calculated.

### 2.5. Confounding Variables Control

To account for potential confounding variables such as comorbidities (e.g., hypertension, diabetes, smoking status), we performed multivariate statistical analyses. Variables identified as statistically significant in simple linear regression were subsequently included in a multiple linear regression model to assess their independent association with the outcome of interest while controlling for potential confounders. Additionally, binomial logistic regression was used to determine the adjusted odds ratios (ORs) for categorical variables, providing a better estimation of the relative risk between the EO-CRC and LO-CRC groups while mitigating the influence of confounding factors.

### 2.6. Adjustment for Multiple Comparisons

To reduce the risk of errors due to multiple comparisons, we applied Bonferroni correction where appropriate, particularly for analyses involving multiple hypothesis tests. This adjustment ensured that the statistical significance threshold remained stringent across multiple comparisons. For key statistical results, we reported 95% confidence intervals (CIs) alongside *p*-values to provide a more comprehensive representation of effect sizes and variability. CIs were calculated for odds ratios (ORs) in binomial logistic regression and for mean differences in *t*-tests and ANOVA analyses.

## 3. Results

**Figure 1:**
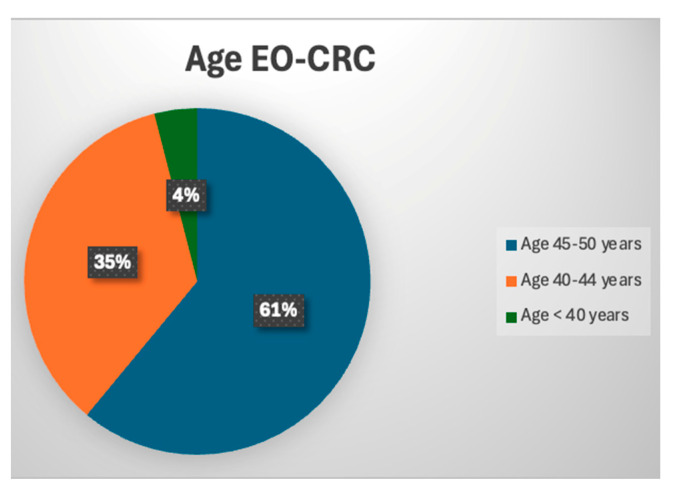
Age group distribution of patients with EO-CRC.

From the entire study group, 23 patients (11.3%) were diagnosed with EO-CRC, while 181 patients (88.7%) had LO-CRC. The mean age of the patients included in our study was 67.76 years, with a minimum age of 31 years and a maximum age of 94 years (Table 1). Regarding the study group distribution, the mean age of patients with EO-CRC was 44.43 years, compared to 70.73 years for those with LO-CRC. The largest proportion of patients with EO-CRC fell within the 45–50 age group (14 patients), followed by the 40–44 age group (8 patients), while only 1 patient was under 40 years old (Figure 1).

**Figure 2:**
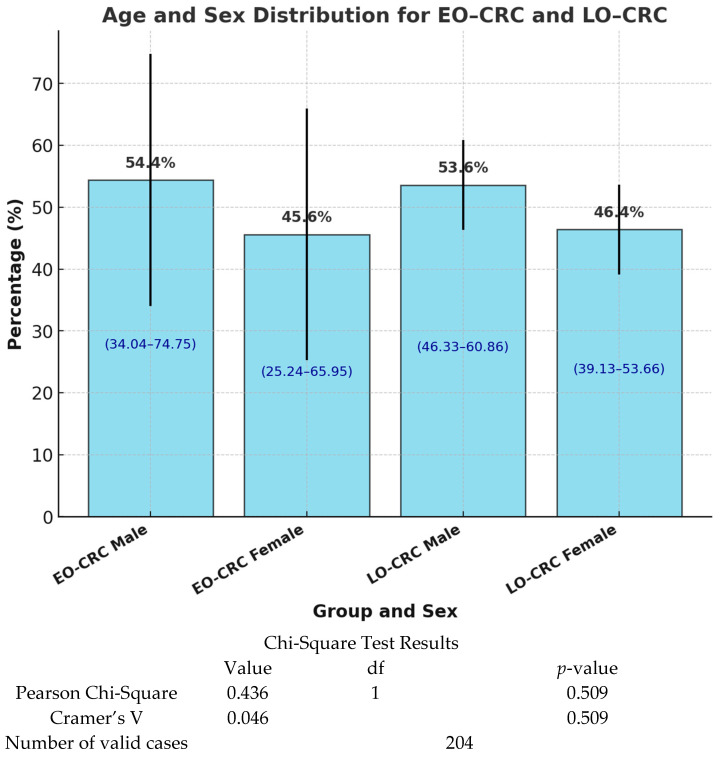
Sex distribution of patients included in the study. This figure illustrates the distribution of patients according to gender, comparing EO-CRC and LO-CRC cases. Data are expressed as percentages (95% CI—95% confidence intervals).

In the total study group, 54.4% of the patients were male, and 45.6% were female. A slight predominance of males was observed in both study groups, and this was more pronounced among patients with EO-CRC. However, these differences were not statistically significant, with a *p*-value of 0.509 (Figure 2).

**Figure 3:**
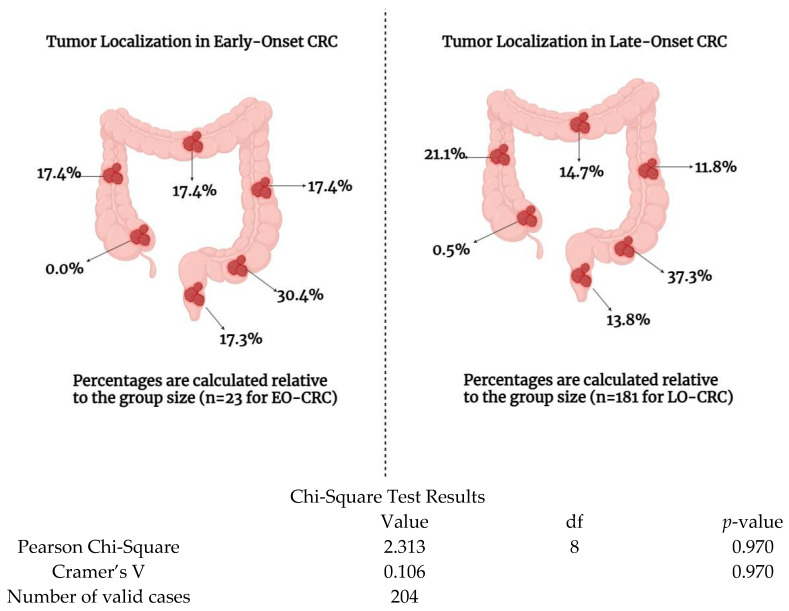
Tumor localization of patients included in the study. This figure illustrates the anatomical distribution of colorectal tumors in patients, comparing early-onset colorectal cancer (EO-CRC) and late-onset colorectal cancer (LO-CRC).

Subsequently, we examined the anatomical location and endoscopic characteristics of the tumors. Although individuals with EO-CRC exhibited a higher prevalence of distal tumor localization and stenotic macroscopic appearance compared to those with LO-CRC, these differences did not reach statistical significance (*p* > 0.05) (Figure 3 and Table 2).

**Figure 4:**
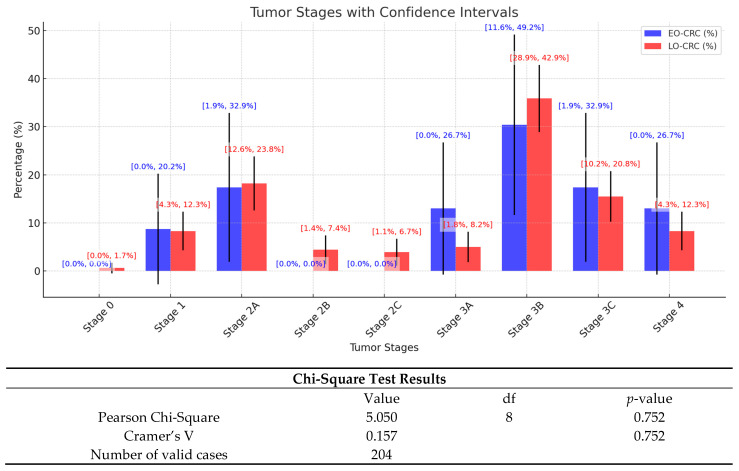
Distribution of patients by tumor stages, presenting 95% confidence intervals (CIs). This figure illustrates the distribution of colorectal cancer stages among patients, comparing EO-CRC and LO-CRC.

We also analyzed the TNM staging and identified a tendency for advanced-stage diagnoses in both patient groups (Figure 4). Thus, stage 4 tumors are more frequent in the EO-CRC patient group compared to LO-CRC, but with a wide confidence interval. Stage 3B was found to be the most common in both groups; however, variability is also higher in the EO-CRC patient group.

**Figure 5:**
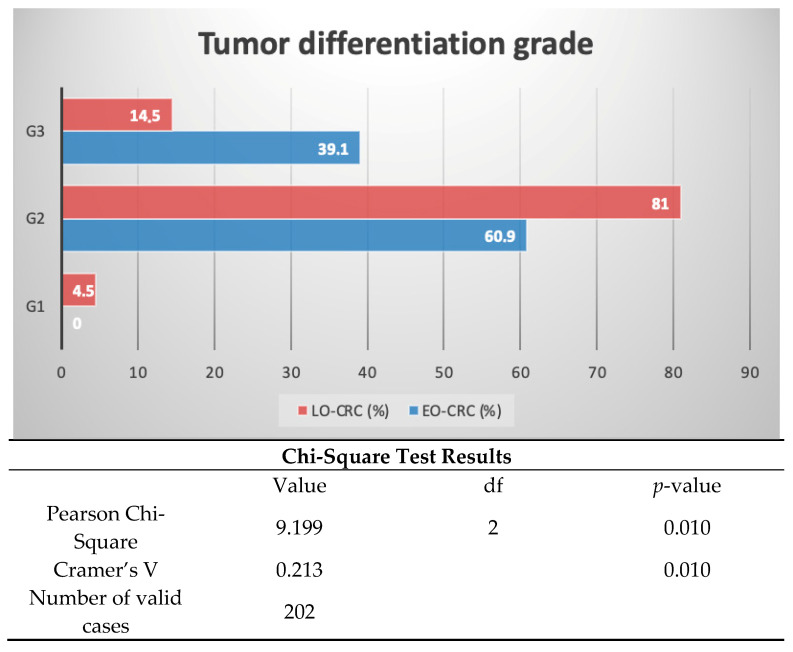
Distribution of patients by tumor differentiation grade. This figure illustrates the distribution of colorectal cancer cases based on tumor differentiation grade, comparing EO-CRC and LO-CRC. Tumor differentiation was categorized as well differentiated (G1), moderately differentiated (G2), and poorly differentiated (G3). Data are presented as percentages, with statistical analysis performed using chi-square test, with a significance threshold set at *p* < 0.05.

Regarding pathological diagnosis and tumor differentiation grade, we noted a greater prevalence of mucinous histological type in patients with EO-CRC compared to those with LO-CRC (34.8% vs. 14.4%). Moreover, poorly differentiated tumors (39.1% G3 vs. 14.2% G3) were much more prevalent in the EO-CRC cohort, with this observation being statistically significant (Table 3 and Figure 5).

The results of the immunohistochemical examination were available for a limited number of patients. However, statistically significant differences were identified in the positivity rates for CDX2 (caudal-type homeobox transcription 2; *p* = 0.012) and HER2 (human epidermal growth factor receptor 2; *p* = 0.002) between the two study groups. Specifically, CDX2 was negative in all EO-CRC patients (for whom immunohistochemical examination was available) and positive in 58.5% of LO-CRC patients (Table 4). In contrast, HER2 was positive in 50% of EO-CRC patients and in none of the LO-CRC patients (Table 5). Additionally, the positivity rate for MSH2 (76.5% in LO-CRC patients versus 33.3% in EO-CRC patients; *p* = 0.056) was at the threshold of statistical significance (Table 4).

In the second part of the study, we comparatively evaluated the prevalence of comorbidities between the two study groups. For this analysis, we initially employed simple binomial logistic regression, where the predictor variables included obesity, smoking, alcohol consumption, inflammatory bowel diseases, abdominal irradiation, cholecystectomy, genetic syndromes, diabetes mellitus, hypertension, renal insufficiency, chronic obstructive pulmonary disease (COPD), family history of cancer, and personal history of cancer at other sites. Among these factors, smoking, hypertension, and a personal history of cancer at other sites were found to significantly increase the risk of EO-CRC in a statistically significant manner (Table 5). For smoking, the odds ratio was 3.450, indicating that smokers have a 3.45-fold higher risk of developing EO-CRC compared to non-smokers (Table 5).

In the next step, the variables with statistical significance from the simple binomial logistic regression analysis were included in a multiple binomial logistic regression analysis. Among these, only smoking and hypertension maintained their statistical significance (Table 6). Additionally, the Wald test values indicate that the regression coefficients βi are significantly different from zero. Consequently, the null hypothesis is dismissed.

Another objective of our study was the comparative evaluation of the serum levels of albumin, carcinoembryonic antigen (CEA), and CA 19-9 between the two study groups. The independent samples *t*-test revealed the absence of significant differences between EO-CRC and LO-CRC patients regarding both CA 19-9 levels (*p* = 0.699) and CEA levels (*p* = 0.956). In contrast, the mean serum albumin levels were found to be significantly higher in patients with EO-CRC compared to those with LO-CRC (*p* = 0.002) (Table 7). Additionally, we sought to identify correlations between the serum levels of the three biomarkers and tumor differentiation grade by using one-way ANOVA (Table 8). However, this analysis did not reveal any statistically significant correlations between the aforementioned variables (Table 8).

The average length of hospital stay for patients included in our study was 14.51 days, with a minimum duration of 1 day and a maximum duration of 60 days. We also analyzed the impact of factors such as age, sex, endoscopic tumor appearance, histopathological diagnosis, tumor differentiation grade, immunohistochemical results, presence of comorbidities, and levels of albumin, CEA, and CA-19-9 on the length of hospital stay, initially using simple linear regression analysis (Table 9). Among the variables mentioned above, the only one that significantly influenced the length of hospital stay was the serum albumin level (*p* = 0.001). The effect size indicator in simple linear regression was represented by r^2^. Table 9 presents the unstandardized regression coefficients (B), standardized coefficients (beta), and the results of the *t*-tests for each of these coefficients. In simple linear regression, beta (standardized coefficient β = −0.507) represents the correlation coefficient between the independent variable and the dependent variable. The unstandardized coefficient for the albumin variable is b = −7.011, which represents the slope of the regression line. The unstandardized coefficient for the constant is a = 37.299, representing the intercept. We can conclude that serum albumin levels may influence the length of hospital stay, depending on its value.

## 4. Discussion

EO-CRC has become a global public health concern, highlighting the need to understand the pathophysiological mechanisms involved in the development of this condition, as well as the implementation of personalized diagnostic and treatment strategies. Based on these considerations, we conducted an observational study aimed at identifying demographic, clinical, and paraclinical differences between patients with EO-CRC and those with LO-CRC.

Of the total study sample, the percentage of patients with EO-CRC was 11.3%. This result is consistent with the data reported in the literature, which estimate that approximately 10% of CRC patients are under the age of 50 [19,20,21]. Wolf et al., in a recent study, report CRC as the most commonly diagnosed cancer and the leading cause of cancer-related death among men under the age of 50 [22].

Most patients with EO-CRC (61%) fell into the 45–50 age group. This validates the data in the literature, which suggest that CRC screening in the 45–50 age group could have a significant impact on patient prognosis [19]. Additionally, screening and endoscopic polypectomy in younger adults may also provide benefits for the 50–55 age group, considering the average progression time from adenoma to carcinoma of approximately 10 years [19,23].

Regarding sex distribution, we identified a slight male predominance in both study groups, which was more pronounced among EO-CRC patients. However, statistical analysis indicates that these differences are not statistically significant. The results reported in the literature are controversial. For instance, Gausman et al. identified male sex as a predictive factor for EO-CRC (odds ratio 1.87), while Ystgaard et al. did not find significant differences in EO-CRC prevalence between sexes [24,25]. Therefore, we conclude that, although EO-CRC appears to be slightly more common in men, patient sex should not influence the screening strategy for this malignancy.

Next, we analyzed the endoscopic and histopathological findings of the patients included in our study. Among EO-CRC patients compared to LO-CRC patients, we observed a tendency toward more distal tumor localization and stenotic endoscopic appearance. Although these results were not statistically significant, they align with findings from the literature. Siegel et al. emphasize that the increased incidence of EO-CRC is largely attributed to higher rates of rectal cancer [15].

In our study, the percentage of rectal tumor localizations was significantly higher in EO-CRC patients compared to LO-CRC patients (17.39% vs. 7.73%). Furthermore, recent studies have demonstrated molecular differences between colonic and rectal cancers, potentially due to divergent exposomal factors [18,26]. For example, BAP1 (BRCA-1 associated protein 1) appears to play a critical role in colonic carcinogenesis but not in rectal cancer [27]. Additionally, in right-sided colon cancer, adenomatous polyposis coli (APC) and tumor protein 53 (TP53) mutations are independent events, whereas in rectal cancer, the key initiating mutations involve Kirsten rat sarcoma virus (KRAS) and TP53 [27].

Regarding the histopathological diagnosis, we identified a higher proportion of the mucinous histologic subtype in EO-CRC patients compared to LO-CRC patients (34.8% vs. 14.4%). Although these results are consistent with those reported in the literature, they were not statistically significant [28,29,30]. This may be explained by the relatively small study cohort. Hashmi et al. reported the presence of the mucinous histologic subtype in 42.6% of EO-CRC patients, emphasizing the more aggressive nature of this cancer type [28]. The higher prevalence of mucinous adenocarcinoma in EO-CRC patients in our cohort aligns with these findings, further reinforcing the distinct molecular signature of EO-CRC compared to LO-CRC.

The majority of CRC patients, regardless of study group, presented with moderately differentiated tumors. However, we identified a significantly higher proportion of poorly differentiated tumors among EO-CRC patients compared to LO-CRC patients (39.1% vs. 14.5%; *p* = 0.010). These findings are consistent with the previously mentioned results and align with the literature [28,31,32,33,34]. For instance, Hashmi et al. reported grade 3 differentiation tumors in 44.1% of EO-CRC patients and only 11.9% of LO-CRC patients [28].

Regarding tumor staging, we did not identify statistically significant differences between the two study groups. This result contrasts with findings reported in the literature, which indicate that EO-CRC patients are more often diagnosed at advanced stages compared to LO-CRC patients [35,36,37,38]. A possible explanation for this discrepancy could be the absence of CRC screening programs in Romania, leading to the frequent diagnosis of these patients at advanced stages, regardless of their age.

Immunohistochemical results were available for a limited percentage of patients. However, we identified statistically significant differences for CDX2 and HER2 expression. CDX2 positivity was more frequent in LO-CRC patients, while HER2 positivity was higher in EO-CRC patients. CDX2 is a gene encoding transcription factors involved in intestinal oncogenesis, and its reduced expression has been linked to more aggressive tumor behavior. Tomasello G et al. demonstrated that CDX2 expression is associated with a 52% reduction in the risk of tumor recurrence and death [39]. Asgari-Karschekani et al. observed that mucinous adenocarcinoma typically exhibits reduced or even negative CDX2 expression, in contrast to conventional adenocarcinoma, where CDX2 positivity is common [40]. In our study, the higher prevalence of mucinous adenocarcinoma in EO-CRC patients aligns with these findings.

The higher HER2 positivity in EO-CRC patients compared to LO-CRC patients is also supported by the literature [28]. HER2 expression in colorectal tumors is associated with poorer differentiation, lymph node metastases, and worse prognosis [28]. Our findings are consistent with these observations, as we demonstrated a significant proportion of poorly differentiated tumors in EO-CRC patients. HER2 overexpression in EO-CRC may drive tumor aggressiveness through activation of key oncogenic pathways, including phosphoinositide 3-kinase (PI3K)/AKT and mitogen-activated protein kinase (MAPK) signaling, which promote cell proliferation, survival, and metastasis [41,42]. Furthermore, HER2 alterations have been linked to resistance to conventional chemotherapy, emphasizing the potential benefit of anti-HER2 targeted therapies in select EO-CRC patients [43].

Given the significant differences in HER2 and CDX2 expression between EO-CRC and LO-CRC patients, stratification of CRC patients based on these biomarkers may have important therapeutic implications. HER2-positive CRC patients could benefit from targeted anti-HER2 therapies, similar to approaches used in HER2-positive breast and gastric cancers [43]. Additionally, the reduced expression of CDX2 in EO-CRC suggests a more aggressive phenotype, which may necessitate closer follow-up and alternative therapeutic strategies. Future research should explore the integration of HER2 and CDX2 status into personalized treatment algorithms for CRC, optimizing therapeutic outcomes and improving patient prognosis.

Another observation from our study is the higher rate of microsatellite instability (MSI) marker positivity in LO-CRC patients compared to EO-CRC patients. Although these results were not statistically significant, they align with findings in the literature [40]. In sporadic CRC, MSI has been strongly associated with BRAF mutations (v-raf murine sarcoma viral oncogene homolog B1), older age, right-sided tumor localization, and high levels of the CpG island methylator phenotype (CIMP) [40].

Our study also provides new insights into the role of comorbidities in EO-CRC, identifying hypertension and smoking as more prevalent in EO-CRC patients. These findings reinforce the need for integrated prevention strategies targeting modifiable risk factors in younger CRC patients. Smoking is a well-known risk factor for CRC [44,45,46]. Recent studies have highlighted its association with advanced adenomas and serrated polyps, which can develop at younger ages and progress to EO-CRC [44,45,46,47].

Regarding the link between hypertension and an increased risk of EO-CRC, we consider this to be primarily related to hypertension as part of the metabolic syndrome. However, obesity has not been shown to be a predictor for EO-CRC. The body mass index (BMI) of all patients in our study was assessed in the context of cancer-related cachexia. Thus, we consider this result non-relevant, as we lacked data on BMI values prior to the cancer diagnosis. Elevated body fat percentage during childhood or adolescence is associated with gut dysbiosis and chronic inflammation, both of which are key mechanisms in colorectal oncogenesis [7,48,49].

Finally, we performed a comparative evaluation of serum levels of CEA, CA19-9, and albumin, identifying only one statistically significant difference: higher albumin levels in EO-CRC patients compared to LO-CRC patients. The clinical relevance of this finding remains unclear, but it may be linked to differences in nutritional status and systemic inflammation. Additionally, we observed a significant correlation between serum albumin levels and hospital stay duration, a finding supported by recent meta-analyses emphasizing the prognostic value of albumin in CRC patients [50,51].

Our study contributes to the growing body of evidence highlighting distinct molecular, histopathological, and clinical characteristics of EO-CRC, reinforcing its aggressive nature and unique biomarker profile. The identification of HER2 positivity and poor tumor differentiation as potential prognostic markers may have implications for personalized treatment approaches in younger CRC patients. Future studies should focus on larger, multicenter cohorts to validate these findings and explore their therapeutic relevance in reducing the mortality rate.

Our study has several limitations, including its retrospective design, small cohort size, and single-center nature, which may impact the generalizability of the findings. The small sample size increases the risk of type II errors, potentially leading to an underestimation of certain associations. Additionally, the single-center design may introduce selection bias and limit the applicability of our results to broader populations. The retrospective nature of the study restricts the ability to establish causality between observed factors and EO-CRC aggressiveness. Future prospective, multicenter studies with larger cohorts are necessary to validate our findings and further elucidate the molecular and clinical characteristics of EO-CRC.

## 5. Conclusions

In conclusion, the alarming increase in EO-CRC incidence rates underscores the need for expanded research in this field, as well as active public awareness campaigns regarding the risk of developing this malignancy. Furthermore, our findings support the consideration of lowering the age for implementing CRC screening strategies in the general population in Europe to 45 years, aligning with the recommendations of American guidelines. However, larger multicenter studies are required to confirm the potential impact of such measures.

Through our study, we highlight the presence of anatomical and molecular differences in colorectal tumors based on patient age. Our findings suggest a more aggressive nature of EO-CRC compared to LO-CRC, potentially explained by the higher frequency of the mucinous histologic subtype, poor tumor differentiation, increased expression of pro-oncogenic factors such as HER2, and reduced expression of tumor suppressor factors such as CDX2. Nevertheless, our study has several limitations that should be considered when interpreting the results. The relatively small sample size and the single-center design may limit the generalizability of our findings. Additionally, the retrospective nature of the study could introduce selection bias. Therefore, further validation in larger, multicenter cohorts is necessary to establish the prognostic significance of these molecular differences.

Additionally, the only biomarkers currently used in the management of CRC patients have demonstrated limited diagnostic value. Under these circumstances, we emphasize the need for future studies to investigate the correlations between exposure to specific risk factors and molecular changes at the tumor level. Future research should focus on prospective validation studies and multicenter collaborations to confirm our findings and assess their clinical significance. Additionally, efforts should be directed towards identifying non-invasive biomarkers for the early diagnosis of EO-CRC and developing personalized therapeutic approaches. These strategies could ultimately lead to improved screening, treatment, and prognosis for patients affected by EO-CRC.

## Figures and Tables

**Table 1:** Age group distribution of patients included in the study. This table presents the distribution of patients according to age groups, distinguishing between EO-CRC and LO-CRC.

Descriptive Statistics						
Age	N		Mean	Standard Deviation	Minimum	Maximum
	Valid Data	Missing Data				
EO-CRC	23	0	44.43	4.541	31	50
LO-CRC	181	0	70.73	8.591	53	94

**Table 2:** Endoscopic appearance of the tumors in patients included in the study (95% CI–95% confidence intervals; df—degrees of freedom). This table presents the distribution of endoscopic tumor features among patients with early-onset colorectal cancer (EO-CRC) and late-onset colorectal cancer (LO-CRC).

		Age					
		EO-CRC			LO-CRC		
		Frequency	%	95% CI	Frequency	%	95% CI
Endoscopic appearance	Infiltrative	1	4.3	(−3.72, 12.28)	17	9.4	(5.01, 13.79)
	Stenosing	10	43.5	(23.16, 63.84)	53	29.3	(22.48, 36.07)
	Ulcerated	11	47.82	(27.23, 68.40)	103	56.91	(49.42, 64.40)
	Vegetative	3	13%	(27.23, 68.40)	42	23.2	(49.42, 64.40)
Total		23	100.0	(−1.57, 27.64)	181	100.0	(16.47, 29.93)
Chi-Square Test Results							
		Value		Df		*p*-value	
Pearson Chi-Square		3.22		3		0.36	
Cramer’s V		0.123				0.36	
Number of valid cases		204					

**Table 3:** Distribution of patients by histological type of tumor formations (95% CI–95% confidence intervals). This table presents the distribution of colorectal tumors based on histological classification, comparing EO-CRC and LO-CRC. Data are expressed as absolute numbers and percentages, with 95% confidence intervals (CIs) provided for each histological subtype.

Age	Histological Type		%	Frequency	95% CI
**EO-CRC**	Mucinous adenocarcinoma		34.8	8	(15.33, 54.26)
	Adenocarcinoma NOS		65.2	15	(40.95, 80.84)
	Invasive adenocarcinoma NOS		4.3	1	(0.00, 12.59)
	Total		100.0	23	
**LO-CRC**	Signet ring cell adenocarcinoma		1.7	3	(0.00, 3.58)
	Mucinous adenocarcinoma		14.4	26	(9.28, 19.51)
	Mucinous adenocarcinoma with signet ring cell component		0.6	1	(0.00, 1.72)
	Adenocarcinoma NOS		81.2	143	(73.06, 84.93)
	Adenocarcinoma NOS with areas of mucinous adenocarcinoma		1.7	3	(0.00, 3.58)
	Invasive adenocarcinoma NOS		0.6	1	(0.00, 1.72)
	Tubular adenocarcinoma		1.1	2	(0.00, 2.61)
	Tubulovillous adenocarcinoma		0.6	1	(0.00, 1.72)
	Carcinoma in situ		0.6	1	(0.00, 1.72)
	Total		100.0	181	
**Chi-Square Test Results**					
Pearson Chi-Square		Value	df	*p*-value	
Cramer’s V		10.373	8	0.240	
Number of valid cases		0.225		0.240	

**Table 4:** Immunohistochemical examination results by study group (CDX2—caudal-type homeobox transcription factor 2; HER2—human epidermal growth factor receptor 2; Ki67—antigen Kiel 67; MLH1—MutL homolog 1; MSH2—MutS homolog 2; MSH6—MutS homolog 6; PMS2—post-meiotic segregation increased 2; AE1/3—cytokeratin AE1/AE3; CA 19.9—carbohydrate antigen 19.9; CK20—cytokeratin 20; CK7—cytokeratin 7; DPC—DNA–protein cross-links; ER—estrogen receptor).

Biomarker	EO-CRC		LO-CRC		Chi-Square Test Results	
	Negative	Positive	Negative	Positive		
CDX2	6 (100%)	0 (0.0%)	7 (41.2%)	10 (58.8%)	Pearson Chi-Square, 6.244 Cramer’s V, 0.521 Validated cases, 23 df 1	*p* 0.012
HER2	3 (50%)	3 (50%)	17 (100%)	0 (0.00%)	Pearson Chi-Square, 9.775 Cramer’s V, 0.652 Validated cases, 23 df 1	*p* 0.002
Ki67	0 (0.0%)	6 (100%)	2 (11.8%)	15 (88.2%)	Pearson Chi-Square, 0.773 Cramer’s V, 0.183 Validated cases, 23 df 1	*p* 0.379
MLH1	4 (66.7%)	2 (33.3%)	5 (29.4%)	12 (70.6%)	Pearson Chi-Square, 2.584 Cramer’s V, 0.335 Validated cases, 23 df 1	*p* 0.108
MSH2	4 (66.7%)	2 (33.3%)	4 (23.5%)	13 (76.5%)	Pearson Chi-Square, 0.829 Cramer’s V, 0.190 Validated cases, 23 df 1	*p* 0.056
MSH6	3 (50%)	3 (50%)	5 (29.4%)	12 (70.6%)	Pearson Chi-Square, 0.829 Cramer’s V, 0.190 Validated cases, 23 df 1	*p* 0.363
PMS2	4 (66.7%)	2 (33.3%)	5 (29.4%)	12 (70.6%)	Pearson Chi-Square, 2.584 Cramer’s V, 0.335 Validated cases, 23 df 1	*p* 0.108
AE1/3	6 (100%)	0 (0.0%)	16 (94.1%)	1 (5.9%)	Pearson Chi-Square, 0.369 Cramer’s V, 0.127 Validated cases, 23 df 1	*p* 0.544
CA 19.9 focal	6 (100%)	0 (0.0%)	16 (94.1%)	1 (5.9%)	Pearson Chi-Square, 0.369 Cramer’s V, 0.127 Validated cases, 23 df 1	*p* 0.544
CK20	6 (100%)	0 (0.0%)	15 (88.2%)	2 (11.8%)	Pearson Chi-Square, 0.773 Cramer’s V, 0.183 Validated cases, 23 df 1	*p* 0.379
CK7	6 (100%)	0 (0.0%)	16 (94.1%)	1 (5.9%)	Pearson Chi-Square, 0.369 Cramer’s V, 0.127 Validated cases, 23 df 1	*p* 0.544
DPC	6 (100%)	0 (0.0%)	16 (94.1%)	1 (5.9%)	Pearson Chi-Square, 0.369 Cramer’s V, 0.127 Validated cases, 23 df 1	*p* 0.544
ER	5 (83.3%)	1 (16.7%)	17 (100%)	0 (0.0%)	Pearson Chi-Square, 2.962 Cramer’s V, 0.359 Validated cases, 23 df 1	*p* 0.085

**Table 5:** Simple binomial logistic regression analysis of the impact of patient comorbidities on the risk of progression to EO-CRC versus LO-CRC. The estimated levels of the regression coefficients βi are denoted by B, and OR represents the “odds ratio” for each factorial variable.

Comorbidity	B	S.E.	Wald	df	*p*-Value	OR
Obesity	−0.396	0.463	0.731	1	0.393	0.673
Smoking	1.238	0.456	7.387	1	0.007	3.450
Alcohol consumption	1.732	0.942	3.383	1	0.066	5.651
Inflammatory bowel diseases	−0.687	1.057	0.422	1	0.516	0.503
Abdominal irradiation	−0.888	1.052	0.712	1	0.399	0.412
Cholecystectomy	0.246	0.665	0.137	1	0.711	1.279
Genetic syndromes	−19.145	40,192.933	0.000	1	1.000	0.000
Diabetes mellitus	1.304	0.759	2.949	1	0.086	3.683
Hypertension	−1.458	0.467	9.755	1	0.002	0.233
Renal insufficiency	−19.191	13,397.653	0.000	1	0.999	0.000
Chronic obstructive pulmonary disease	−19.197	12,710.136	0.000	1	0.999	0.000
Family history of cancer	0.992	1.177	0.711	1	0.399	2.697
Personal history of cancer at other sites	−1.389	0.638	4.743	1	0.029	0.249

**Table 6:** Multiple binomial logistic regression analysis of the impact of patient comorbidities on the risk of progression to EO-CRC versus LO-CRC. The estimated levels of the regression coefficients βi are denoted by B, and OR represents the “odds ratio” for each factorial variable. The odds ratio is an estimate of the “risk” of a subject achieving a positive clinical response with a one-unit change in the factorial variables. Variable (s) entered on step 1: hypertension; variable (s) entered on step 2: smoking.

Variables in Equation							
Step 1	Hypertension (1)	**B**	**S.E.**	**Wald**	**df**	***p***	**OR**
	Constant	−1.458	0.467	9.755	1	0.002	0.233
Step 2	Smoking (1)	2.757	0.365	57.171	1	0.000	15.750
	HTA (1)	1.321	0.477	7.678	1	0.006	3.749
	Constant	−1.523	0.480	10.048	1	0.002	0.218

**Table 7:** Comparative analysis of tumor biomarkers and albumin levels using independent samples *t*-test.

		Levene’s Test for Equality of Variances		*t*-Test for Equality of Means				
		F	*p*-Value	t	df	*p*	Cohen’s d	F
CEA (ng/mL)	Equal variances assumed	0.133	0.716	−0.056	63	0.956	−0.006	0.133
	Equal variances not assumed			−0.095	24.039	0.925		
CA 19-9 (U/mL)	Equal variances assumed	0.372	0.544	−0.389	57	0.699	−0.050	0.372
	Equal variances not assumed			−0.710	34.595	0.482		
Albumin (g/dL)	Equal variances assumed	0.023	0.879	3.092	202	0.002	0.216	0.023
	Equal variances not assumed			3.198	28.455	0.003		

**Table 8:** One-way ANOVA analysis of tumor biomarkers and albumin levels by tumor differentiation grade (well differentiated—G1; moderately differentiated—G2; poorly differentiated—G3).

	Tumor Differentiation Grade	N	Mean	Std. Deviation
CEA (ng/mL)	G1	3	10.23	8.891
	G2	54	26.16	78.384
	G3	8	37.91	41.918
	Total	65	26.87	72.879
CA 19-9 (U/mL)	G1	G1	2	0.989
	G2	G2	50	185.404
	G3	G3	7	48.682
	Total	Total	59	171.475
Albumin (g/dL)	G1	8	3.45	0.615
	G2	157	3.21	0.632
	G3	34	3.30	0.65311
	Total	199	3.24	0.63431
ANOVA				
	F	Eta-Squared (η^2^)		*p*-value
CEA (ng/mL)	0.168	0.005		0.846
CA 19-9 (U/mL)	0.113	0.004		0.894
Albumin (g/dL)	0.759	0.007		0.470

**Table 9:** Simple linear regression analysis—predictors of length of hospital stay (unstandardized regression coefficients B, standardized coefficients beta).

Predictor	B	Std. Error	Beta	t	*p*-Value
Age	0.017	0.053	0.023	0.322	0.748
Sex	−0.324	1.243	0.018	−0.261	0.794
Endoscopic appearance of the tumor	−0.190	0.213	−0.062	−0.889	0.375
Tumor differentiation grade	−0.357	1.426	−0.018	−0.250	0.803
Immunohistochemical examination	1.161	1.995	0.041	0.582	0.561
Inflammatory bowel disease	3.109	2.293	0.095	1.356	0.177
History of abdominal irradiation	−4.102	2.111	−0.135	−1.943	0.053
Cholecystectomy	−2.507	1.988	−0.088	−1.261	0.209
Genetic syndromes	−3.527	8.862	−0.028	−0.398	0.691
Diabetes mellitus	−1.907	1.443	−0.093	−1.321	0.188
Hypertension	−0.398	1.304	−0.021	−0.305	0.760
Renal insufficiency	4.697	2.997	0.110	1.567	0.119
Chronic history of cancer at other site	−3.165	2.859	−0.078	−1.107	0.270
History of cancer at another site	−1.560	1.295	−0.084	−1.204	0.230
Family history of cancer	0.245	4.466	0.004	0.055	0.956
Obesity	0.442	1.250	0.025	0.354	0.724
Smoking	−0.349	1.449	−0.017	−0.241	0.810
Alcohol consumption	0.913	4.004	0.016	0.228	0.820
Albumin	−7.011	0.839	−0.507	−8.355	0.001
CEA	0.003	0.015	0.029	0.229	0.819
CA 19.9	0.002	0.007	0.038	0.287	0.775

## Data Availability

The data that support the findings of this study are available from the corresponding author upon reasonable request.
